# Medial septum tau accumulation induces spatial memory deficit via disrupting medial septum–hippocampus cholinergic pathway

**DOI:** 10.1002/ctm2.428

**Published:** 2021-05-28

**Authors:** Dongqin Wu, Di Gao, Haitao Yu, Guilin Pi, Rui Xiong, Huiyang Lei, Xin Wang, Enjie Liu, Jinwang Ye, Huilin Yu, Yang Gao, Ting He, Tao Jiang, Fei Sun, Jingfen Su, Guoda Song, Wenju Peng, Ying Yang, Jian‐Zhi Wang

**Affiliations:** ^1^ Department of Pathophysiology, School of Basic Medicine, Key Laboratory of Education Ministry of China/Hubei Province for Neurological Disorders, Tongji Medical College Huazhong University of Science and Technology Wuhan China; ^2^ Co‐innovation Center of Neuroregeneration Nantong University Nantong China

**Keywords:** cholinergic neuron, donepezil, medial septum, Tau accumulation

## Abstract

Tau accumulation and cholinergic impairment are characteristic pathologies in Alzheimer's disease (AD). However, the causal role of tau accumulation in cholinergic lesion is elusive. Here, we observed an aberrant tau accumulation in the medial septum (MS) of 3xTg and 5xFAD mice, especially in their cholinergic neurons. Overexpressing hTau in mouse MS (MS^hTau^) for 6 months but not 3 months induced spatial memory impairment without changing object recognition and anxiety‐like behavior, indicating a specific and time‐dependent effect of MS‐hTau accumulation on spatial cognitive functions. With increasing hTau accumulation, the MS^hTau^ mice showed a time‐dependent cholinergic neuron loss with reduced cholinergic projections to the hippocampus. Intraperitoneal administration of donepezil, a cholinesterase inhibitor, for 1 month ameliorated the MS‐hTau‐induced spatial memory deficits with preservation of MS–hippocampal cholinergic pathway and removal of tau load; and the beneficial effects of donepezil was more prominent at low dose. Proteomics revealed that MS‐hTau accumulation deregulated multiple signaling pathways with numerous differentially expressed proteins (DEPs). Among them, the vacuolar protein sorting‐associated protein 37D (VP37D), an autophagy‐related protein, was significantly reduced in MS^hTau^ mice; the reduction of VP37D was restored by donepezil, and the effect was more significant at low dose than high dose. These novel evidences reveal a causal role of tau accumulation in linking MS cholinergic lesion to hippocampus‐dependent spatial cognitive damages as seen in the AD patients, and the new tau‐removal and autophagy‐promoting effects of donepezil may extend its application beyond simple symptom amelioration to potential disease modification.

## Graphical abstract

**Proposed working model**: Medial septum (MS) tau accumulation disrupts MS–hippocampus cholinergic pathway and impairs hippocampus‐associated spatial memory as seen on patients with Alzheimer's disease (AD). In addition to traditional cholinesterase inhibition, low‐dose donepezil exerts novel tau‐removal and autophagy‐promoting effects, efficiently rescuing MS‐htau‐induced memory loss. Our results supplement tau toxicity and the new findings on donepezil may extend its application beyond simple symptom amelioration to potential disease modification; the latter may help physicians to optimize dosing in donepezil treatment on AD.

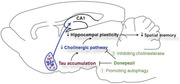

## 1 INTRODUCTION

Alzheimer's disease (AD), an irreversible and currently incurable neurodegenerative disease, is characterized neuropathologically by beta‐amyloid (Aβ) plaques and neurofibrillary tangles (NFTs).^1^, ^2^, ^3^, ^4^ The cholinergic basal forebrain (CBF) projection neurons located within the medial septum (MS) and diagonal band Broca (DDB) are severely damaged along with the aggravation of Aβ and NFTs during AD progression.^5^ By in vivo magnetic resonance imaging, atrophy of MS and DDB have been detected in prodromal AD individuals and demented AD patients.^6^ The reduction of cholinergic neurotransmitter in the cortex and hippocampus, the main target regions of cholinergic innervation from MS and DDB, is correlated with cognitive decline and AD severity.^7^, ^8^, ^9^ Basal forebrain cholinergic neurons degeneration is considered to be the main cause for the decrease of cholinergic projections and neurotransmission. By increasing the amount of acetylcholine within synaptic cleft, cholinesterase inhibitors have been found beneficial in ameliorating AD symptoms.^10^, ^11^, ^12^ However, the causal factor that triggers MS cholinergic deficits is unclear.

At early stage of AD, tau preferentially accumulates in the excitatory neurons,^13^ resulting in the dysfunction of their excitability^13^, ^14^ and synaptic plasticity.^15^ Tau accumulation is also detected in GABAergic interneurons of hippocampal dentate gyrus (DG), where tau impairs hippocampal neurogenesis and increases neural stem cell‐derived astrogliosis.^16^ In human‐induced forebrain cholinergic neurons (hiBFCNs) from skin fibroblasts of sporadic AD patients, tau hyperphosphorylation was developed in a time‐dependent manner. Simultaneously, nucleocytoplasmic transport dysfunction was detectable.^15^, ^17^ Moreover, the acetylcholinesterase (AChE) was found to colocalized with hyperphosphorylated tau (P‐tau) within neurofibrillary tangles in AD. Increasing tau phosphorylation was found to raise AChE activity and protein expression, consequently decreasing neurotransmitter acetylcholine in SH‐SY5Y cells.^18^ In Meynert nucleus basalis, dysregulation of histone deacetylase (HDAC) 2 was correlated with NFT formation in cholinergic neurons.^19^ However, it is unknown whether and how tau may impair MS cholinergic neurons in vivo and thus affect spatial learning and memory, to date.

Based on the current studies, we speculate that the MS tau accumulation may impair the in situ cholinergic neurons and their projections to the hippocampus, thus causes cognitive dysfunction. We tested this hypothesis by overexpressing hTau in the MS subset of mice. We found that hTau accumulation induced a time‐dependent cholinergic neuron loss in MS and their projections to the hippocampus with a simultaneous spatial memory deficit. Intraperitoneal administration of donepezil, a cholinesterase inhibitor, for 1 month improved cognitive capacity and rescued cholinergic loss. Interestingly, donepezil induced a significant tau reduction and the effect of low‐dose donepezil was more significant than high dose. Proteomic analysis revealed that MS‐hTau accumulation deregulated multiple proteins and signaling pathways. Among them, the vacuolar protein sorting associated protein 37D (VP37D), an autophagy‐related protein, was significantly reduced in MS^hTau^ mice, and donepezil treatment restored VP37D level and low‐dose donepezil even increased VP37D level.

## 2 MATERIALS AND METHODS

### 2.1 Animals

3xTg (Stock No: 34830, 129S4.CgTg [APPSwe, tauP301L] 1LfaPsen1tm1Mpm/Mmjax) mice and wild‐type mice were a gift from Prof. Xifei Yang (Shenzhen Center for Disease Control and Prevention). 5xFAD (Stock No: 034840, B6SJL‐Tg [APPSwFlLon, PSEN1*M146L*L286V] 6799Vas/Mmjax) mice were a gift from Prof. Bai Lu (School of Medicine, Tsinghua Univ., Beijing, China). Mice genotypes were identified by PCR. Adult C57BL/6 mice (male, 6–8 weeks) were purchased from Hua Fu Kang Biotechnology Company or Vital River Animal Technology Company in Beijing.

All mice were group housed (four to five) in a dedicated husbandry facility at steady temperature (23–25°C) and humidity (45–60%) under a fixed circadian rhythm (lights on: 7:00 a.m. to 19:00 p.m., lights off: 19:00 p.m. to 7:00 a.m.) with enough water and food. 3xTg and 5xFAD (10‐month old) were used for Western blot and immunostaining, and wild‐type mice (10‐month old) were used for control. C57BL/6 mice (male, p56–84) were randomly divided into groups and given viral injection. The age of the animals in subsequent experiments was marked accordingly. In addition to the accordance with the Tongji Medical College Guide for the Care and Use of Laboratory Animals, all animal experiments were conducted with the approval of Institutional Animal Care and Use Committee of Huazhong University of Science and Technology.

### 2.2 Antibodies, reagents, and viruses

Anti‐pT205 (SAB,11108‐2), anti‐pT231 (SAB,11110), anti‐pS396 (SAB,11102), anti‐pS199 (Invitrogen, 44734G) and anti‐human PHF‐tau (AT8) (Thermo, MN1020), anti‐HT7 (Thermo, MN1000), anti‐Tau5 (Abcam, ab80579) and β‐actin (Abcam, ab6276), NeuN (CST,12943), ChAT (Chemicon, AB144P), Nissl staining solution (Beyotime, C0117), DAB‐staining kit (ZLI‐9031, ZSGB‐BIO), and donepezil hydrochloride (MedChemExpress, E2020) were obtained and used in the present study.

The virus AAV‐CAG‐hTau‐mCherry‐3flag and AAV‐CAG‐vector‐mCherry‐3flag were constructed and packaged based on the plasmid mCherry‐tau‐2N4R and mCherry‐vector‐2N4R, encoding hTau as described in previous study.^15^ The target gene was human microtubule‐associated protein tau (hTau) with gene ID: 4137. The serotype of the virus was AAV8, which has been generally employed in the nervous system.

### 2.3 Stereotaxic surgery

Mice were anesthetized with pentobarbital sodium (1%, 30 mg/kg) and immobilized in a stereotactic injection apparatus (RWD 68046 and 68055, China). After the scalp was disinfected, a middle skin incision was made over the bregma. After balancing the head, a tiny hole was punched very gently at MS (anteroposterior [AP] +0.86 mm, mediolateral [ML] 0 mm, dorsoventral [DV] −4.15 mm). The virus was injected into the MS with a 10‐μl gas‐tight miscrosyringe (KF019, China) under a stereotaxic instrument (World Precision Instruments, USA). The pAAV‐CAG‐hTau‐mCherry‐3flag (0.8 μl, 1.27 × 10^13^ VG/ml) was injected into the MS at a rate of 100 nl/min and the same volume of pAAV‐CAG‐vector‐mCherry‐3flag (1.59 × 10^13^ VG/ml) was injected as control. Lincomycin lidocaine gel was evenly applied to the skull and dissolvable stitches were used to suture the skin. The mice were put on a heated blanket waiting to wake‐up. At the end, the accuracy of the infection site was confirmed by mCherry, and the mice with incorrect mCherry location were excluded.

**HIGHLIGHTS**

1. Tau accumulation in medial septum plays a causal role in linking cholinergic lesion to hippocampus‐associated spatial memory loss.
2. Donepezil treatment exerts novel tau‐removal and autophagy‐promoting effects.
3. Low‐dose donepezil is more efficient than high dosage in removing accumulated tau and rescuing medial septum tau‐induced spatial memory loss.

### 2.4 Drug treatment

Donepezil hydrochloride (MedChemExpress, E2020) was dissolved in normal saline (NS, 0.9% NaCl) as 10 mg/ml stock solution and was stored at −20°C. The Mice were divided into four experimental groups as follows: vector group treated with vehicle (Vec:veh), hTau mice treated with vehicle (hTau:veh), hTau mice treated with donepezil at low dose (1.0 mg/kg) (hTau:DZ[L]) and high‐dose (2.0 mg/kg) (hTau:DZ[H]), respectively. The NS and donepezil were administrated daily through intraperitoneal injection for 4 weeks, started at 5 months after the brain infusion of the AAV virus. All solutions were freshly prepared before daily injection.

### 2.5 Immunostaining

The brain tissue was postfixed in 4% PFA at 4°C for 24 h, and then switched to 25% and 30% sucrose solutions in turn for at least 2 days. Brains were sectioned on a cryogenic frozen microtome (CM1860, Leica, Germany) at the thickness of 30 μm, and stored in the anti‐freeze solution at −20°C for use. Images were obtained by using a laser scanning confocal microscope (LSM710 or LSM780, Zeiss) or virtual slide microscope (SV120, Olympus).

For immunofluorescence staining, the free‐floating sections were washed with PBST (PBS with 0.1% Triton‐100) and permeabilization done with 0.5% Triton‐100 PBS for 20–30 min. QuickBlock blocking buffer (P0260, Beyotime) was used for blocking. Then, the sections were incubated with primary antibody (1:200∼1:500, P0023A) at 4°C overnight and incubated with secondary antibodies (Invitrogen, A11055 or A11081 or A‐31573) for 1–2 h at 37°C in dark (1:500, P0265). Lastly, the sections were counterstained with DAPI and mounted with PBS containing 50% glycerol (pH 7.2) for imaging.

For immunohistochemical staining, the brain sections were incubated with 0.3% H_2_O_2_ (in 0.5% Triton‐100 PBS) at 37°C for 20–30 min before blocking to eliminate endogenous peroxidase activity. The operations for the primary antibody were the same as floating sections. The secondary antibody (AD048/AD049) and ternary antibody (AD050) or hypersensitive ready‐to‐use goat two‐step detection kit (PV‐9003, ZSGB‐BIO) and DAB‐staining kit (ZLI‐9031, ZSGB‐BIO) were used for horseradish peroxidase reaction staining. Then the brain sections were sealed with neutral balsam after being dehydrated through graded ethanol series and hyalinized by dimethylbenzene.

### 2.6 Nissl staining

The brain slices were rinsed in 0.1% Triton 100‐PBS and mounted on adhesive microscope slides to dry before incubated with Nissl Staining Buffer (C0117, Beyotime) for 10 min at 37°C. Then the slices were washed in ddH_2_O quickly, immersed in 95% ethanol for 5 min twice, and soaked in 100% ethanol for 5 min for dehydration. Subsequently, the slices were hyalinized in xylene. All slices were covered with diluted neutral gum. The images were acquired with the automatic section scanning system (Olympus SV120, Tokyo, Japan).

### 2.7 Behavioral tests

All behavioral tests were performed as described previously.^15^, ^16^, ^20^ Before behavioral tests, mice were handled for 2 min per day for at least 3 consecutive days, and the mice were transferred to the behavior detection room about 2 h before the test to adapt to the environment.

For open‐field test, 30% of the open‐field area was defined as central zone. Each mouse was allowed to explore the open‐field area for 5 min freely. At the end of each trial, the area was cleaned with 75% alcohol. The distance moved and the time spent in the central zone were recorded by a camera and analyzed using a software (Chengdu Taimeng Software Co. Ltd, China).

The object–place recognition test was carried out by using the same equipment as for open‐field test. In the habituation session, mice were put in the chamber to explore for 5 min individually. During training day, two similar‐sized objects with diverse shape and color were settled in different corners of the area (2 cm from the wall), and the mice were put in the center and allowed to move freely in the objects‐located area for 5 min. After 24 h, one of the objects was replaced by a similar but different object (novel). The mice were put in the center and allowed to play with the two fixed objects for 5 min. Both the chamber and objects were wiped with 75% ethanol between each trial. The trajectory and related parameters were recorded and analyzed (Chengdu Taimeng Software Co. Ltd). The exploring time for different objects was measured.

The elevated plus maze (EPM) contains an open arm (66 × 6 cm) and a closed arm (66 × 6 cm) with a central area (6 × 6 cm). Each mouse was put in the center area and was allowed to explore for 5 min freely. The maze was wiped with 75% ethanol at the experimental interval. The movement of the mouse was recorded and analyzed by software (Chengdu Taimeng Software Co. Ltd). The time spent in the open arm and the entering times in the open arm were counted, respectively.

Morris water maze (MWM) consisted of a circular pool (diameter: 1.4 m) surrounded by various visual cues on the wall with opaque water at 23–25°C and it was refreshed every day. For spatial learning trial, a cylindrical escape platform (diameter: 10 cm) was placed 1 cm below the water at a fixed position (hidden). The mice were trained to find the platform in the maze, practiced three times a day with 30 min intervals for 5 consecutive days. The trial was started when the mice faced a settled place of the pool of each quadrant and were gently lowered into the water. When the mice climbed up the hidden platform within 60 s, the trail was ended and the mice were kept on the platform for 30 s. If the mice could not find the platform within 60 s, they were guided to the hidden platform and stayed there for 30 s. The movements and the latency to find the hidden platform were automatically recorded. Forty‐eight hours after the last learning trial, spatial memory test was conducted. Mice were allowed to search for 60 s freely while the hidden platform was removed. The escape latency, platform crossing times, time spent in target quadrant, and the distance moved were recorded with the camera and behavior analysis system (Chengdu Taimeng Software Co. Ltd).

### 2.8 Western blotting

Western blotting was performed as described in previous study.^16^ The MS subset was dissected and sliced by oscillating section (Campden 7000 smz, UK) on ice‐cold artificial cerebrospinal fluid (aCSF). The aCSF consists of components including 126 mM NaCl, 24 mM NaHCO_3_, 2 mM MgSO_4_, 3 mM KCl, 1.25 mM NaH_2_PO_4_, 2 mM CaCl_2_, and 10 mM glucose. The total extract was prepared by lysing the MS tissue in RIPA buffer (weak, Beyotime, Shanghai, China) containing 150 mM NaCl, 50 mM Tris (pH 7.4), 0.25% sodium deoxycholate, and 1% NP‐40 with the protease inhibitor cocktail, and then centrifuged at 8000 × *g* for 10 min at 4°C. For preparation of soluble and insoluble tau, the MS tissues were extracted with RIPA (strong, Beyotime, Shanghai, China) containing 0.1% SDS, 1% Triton X‐100, 50 mM Tris (pH 7.4), 1% sodium deoxycholate, and 150 mM NaCl with the protease inhibitor cocktail and then centrifuged at 12,000 × *g* for 15 min at 4°C. The supernatant was defined as soluble tau fraction. The insoluble fraction was prepared by redissolving the pellet with RIPA strong buffer (8% SDS), boiled for 10 min, and bath sonicated 20 times. The protein levels were quantified by using BCA method (Prod# 23224, Thermo, USA) before use.

10% SDS/PAGE was used to separate the proteins and then the proteins were transferred onto nitrocellulose membranes (Whatman). Then, the membranes were blocked with Western blocking buffer (P0023B, Beyotime) for 20–30 min at room temperature and incubated with primary antibody at 4°C overnight. After incubation with the secondary antibody (1:1000; Beyotime, A0181 or A0216 or A0208) at room temperature for 1 h, the membranes were visualized with ECL system (ChemiScope 6000,Shanghai, China).

### 2.9 Golgi‐cox staining

The solution was prepared according to the previous method.^21^ The brains were dipped into Golgi solution for 4–6 weeks in dark with change of fresh solution once after first 24 h. Then the brains were transferred into the tissue protection solution for 7 days at 4°C in dark with change of fresh solution once after first 24 h. The brains were then sectioned by a Vibratome (Leica VT1000, Germany) at 100‐μm thickness and attached to gelatin‐coated glass slides to dry. After 72 h, brain slices were stained in the following sequence: ddH_2_O twice (5 min each), 50% ethanol (5 min), 3:1 ammonia solution (8 min), ddH_2_O twice (5 min each), 5% sodium thiosulfate in dark (10 min), ddH_2_O twice (1 min each), 75%, 90%, 95%, and 100% ethanol (6 min each), Xylol (6 min). At last, the slices were mounted with xylene‐diluted neutral gum. The images were taken using Olympus SV120 (Tokyo) and Nikon Ni‐E (Tokyo).

### 2.10 Electrophysiological recording

After being anesthetized, the mice were sacrificed and the brain were moved into ice‐cold aCSF (pH 7.2–7.4; 300 mOsm) saturated with carbogen (95% O_2_ and 5% CO_2_). The hippocampus slice (300 mm) were prepared with Vibratome (Leica VT1000, Germany) and incubated at 25°C in aCSF for 60 min. Then, the slices were transferred into a chamber with 64 microelectrode arrays (each 50 × 50 μm in size) and maintained with circulating aCSF perfusion (1–2 ml/min). The MED64 electrophysiological recording system (MED‐PA5455, Panasonic) was used to acquire signals. The field excitatory postsynaptic potentials (fEPSPs) were recorded in CA1 neurons by stimulating the CA3 Schaeffer fibers. Maximal size stimulating intensity of 30% was applied to induce fEPSP and three trains of high‐frequency stimulation (HFS, 100 Hz, 1 s duration) were used to evoke long‐term potentiation (LTP).

### 2.11 Proteomic analysis

For sample preparation, the MS^hTau^ mice (with MS hTau overexpression for 6 months) and the control mice with or without donepezil administration were euthanized and decapitated. The brain was cut (300 mm) with Vibratome (Campden 7000smz, UK) in ice‐cold aCSF. The MS subset was dissected on ice by referring to the atlas of the mouse brain (the third edition). Then the MS tissue was lysed in 8 mol/L urea buffer and ultrasonized. The protein concentration was acquired by nanodrop 2000C (Thermo Scientific, USA).

After the protein digestion (100 μg), samples were divided into groups, labeled with TMT (Thermo Fisher 90406), and separated by following the instruction for LC‐MS/MS system. By using data‐dependent acquisition mode, the top 10−20 data‐dependent MS/MS scans were carried out with full scans in Orbitrap mass analyzer.^22^ The MS/MS spectra were searched using SEQUEST search algorithms embedded into Proteome Discoverer 2.1 (Thermo Scientific) against the Uniport mouse database (downloaded from Uniport database on 2020‐5). The searching parameters were set to the modified default value. All raw data were uploaded to the Proteome Xchange Consortium with the data identifier PXD023297.

The abundance of all proteins was normalized by Perseus computational platform, and the differentially expressed proteins (DEPs) were obtained after *t*‐test (requirements: adjusted *p* < .05, fold change >1.2 or <0.83). R studio (v. 0.99.489) and heatmap package were used for cluster analysis and heatmap drawing.

### 2.12 Statistical analysis

GraphPad Prism (version 8.0) was used for statistical analyses. Student's *t*‐test was used for two‐group comparisons. One‐way ANOVA or two‐way ANOVA followed by post hoc tests was conducted for multiple comparisons. All data were presented as mean ± SEM; *p* < .05 was considered as statistically significant.

## 3 RESULTS

### 3.1 Increased tau accumulation with cholinergic neuron loss detected in MS of 10‐month‐old 3xTg and 5xFAD mice

**FIGURE 1:**
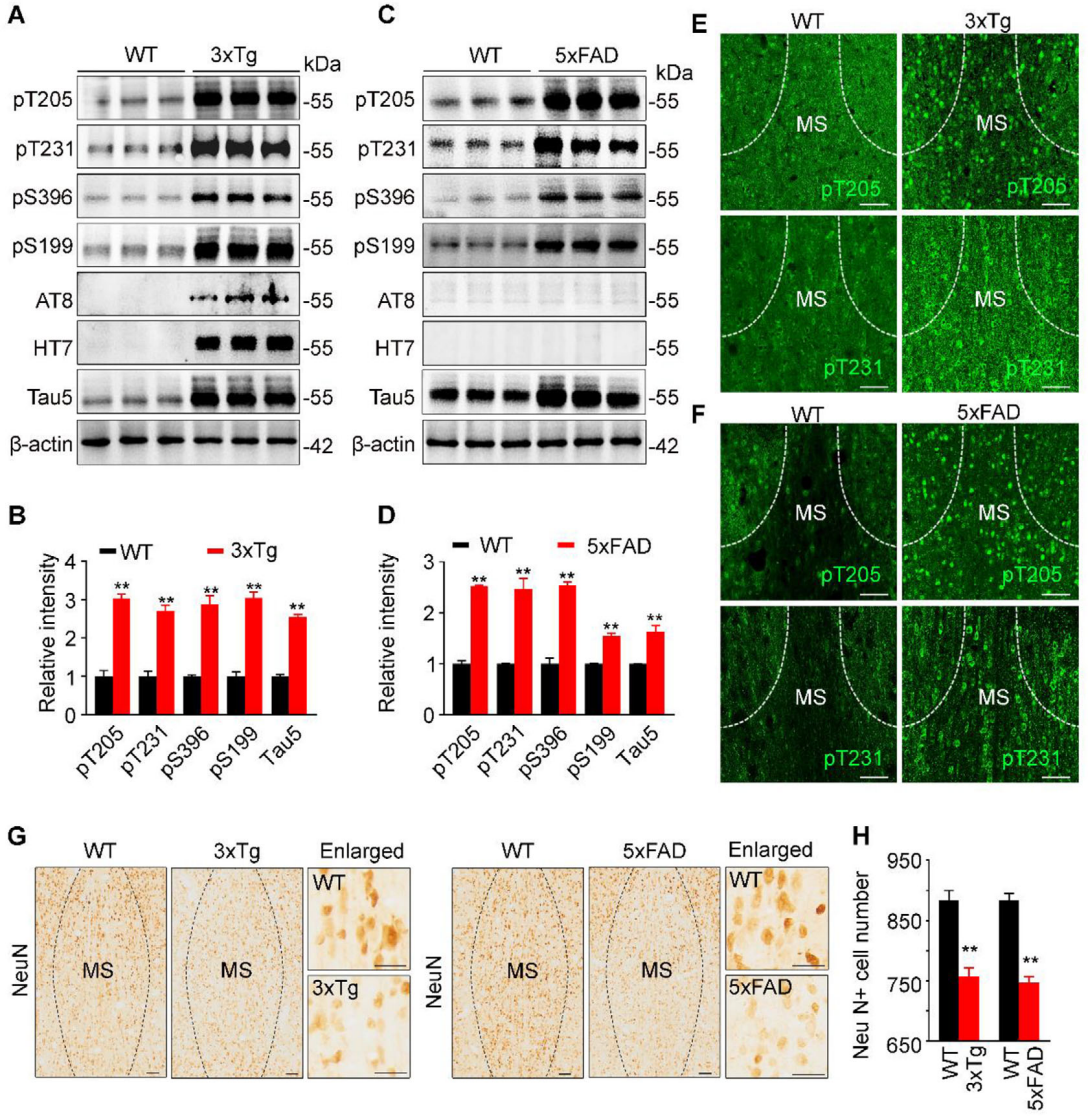
Accumulation of hyperphosphorylated tau and neuron loss in the medial septum (MS) of 3xTg and 5xFAD mice. (A–D) Hyperphosphorylated tau (pT205, pT231, pS396, pS199, AT8) and total tau (Tau5) were increased in MS subset of 10‐month‐old 3xTg (A, B) and 5xFAD (C, D) mice compared with age‐ and sex‐matched wild‐type mice measured by Western blotting. HT7 reacts specifically with human tau, and AT8 preferentially probes pathologically hyperphosphorylated tau proteins. *n* = 6 Mice per group. For 3xTg versus control, unpaired *t‐*test, [pT205] *t* = 10.73 df = 4, *p *= .0004; [pT231] *t* = 8.245 df = 4, *p *< .01; [pS396] *t* = 8.204 df = 4, *p *< .01; [pS199] *t* = 10.8 df = 4, *p *< .01; [Tau5] *t* = 21.15 df = 4, *p *< .01. For 5xFAD versus control, unpaired *t‐*test, [pT205] *t* = 22.80 df = 4, *p *< .01; [pT231] *t* = 6.939 df = 4, *p *< .01; [pS396] *t* = 11.38 df = 4, *p *< .01; [pS199] *t* = 10.95 df = 4, *p *< .01; [Tau5] *t* = 5.316 df = 4, *p *< .01. (E and F) Prominent accumulation of phosphorylated tau (pT231 and pT205) in the MS of 3xTg (E) and 5xFAD (F) mice measured by immunofluorescence staining. Scale bar, 100 μm. (G and H) Significant neuron loss in MS of 3xTg and 5xFAD mice measured by NeuN staining. Scale bar, 100 μm or 50 μm (in enlarged images). *n* = 3 Mice per group. Unpaired *t*‐test, for 3xTg versus control, *t* = 5.705 df = 46, *p *< .01; for 5xFAD versus control, *t* = 9.250 df = 46, *p *< .01. *p < .05, **p < .01 versus WT group. Data are presented as mean ± SEM

To explore whether MS is susceptible to tau pathology in AD process, we employed two AD mouse models, 3xTg AD mice and 5xTg AD mice, to detect tau expression and distribution in MS. The age‐ and sex‐matched wild‐type mice were used as controls. By precisely dissected MS subset, we found that levels of total tau probed by Tau5 and the phosphorylated tau at multiple AD‐associated sites, including pT205, pT231, pS396, pS199, and AT8 epitope (pS202/pT205), were significantly increased in 3xTg AD mice (Figure 1A,B). In 5xFAD mice, increased total tau and phosphorylated tau at pT205, pT231, pS396, and pS199 were also detected (Figure 1C,D). The positive signal of HT7, which specifically reacts with human tau, was only detected in 3xTg AD mice (Figure 1A,C). By immunofluorescent staining, the increased phospho‐tau at pT205 and pT231 was also detected in both AD mouse models, and the hyperphosphorylated tau proteins were robustly accumulated in the nucleus (pT205) and the cell body (pT231), respectively (Figure 1E,F). By immunohistochemistry staining, significant neuron loss was also detected in MS of 10‐month‐old 3xTg and 5xFAD mice (Figure 1G,H).

**FIGURE 2:**
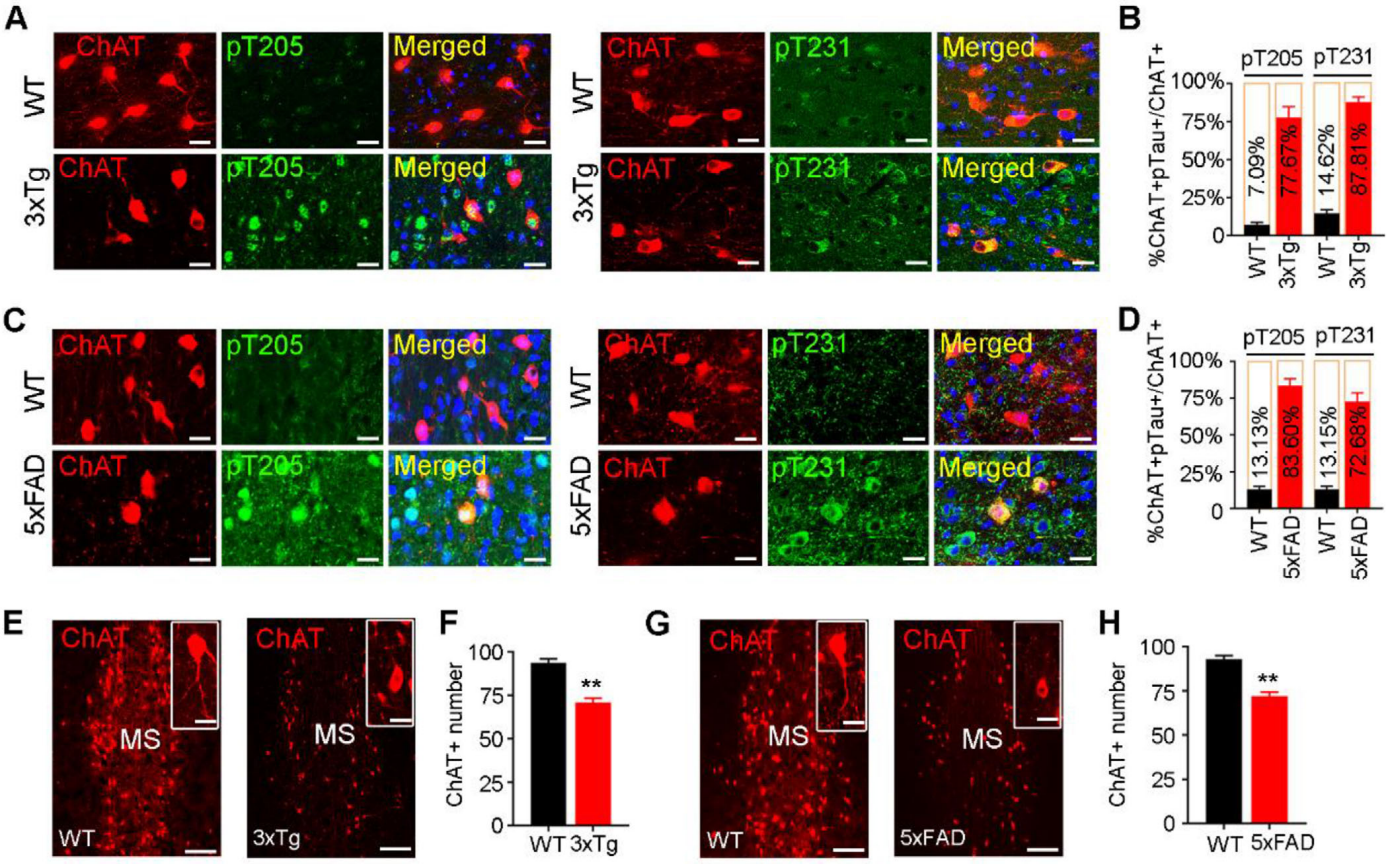
Accumulation of phosphorylated tau correlates with cholinergic neuron loss in MS of 3xTg and 5xFAD mice. (A–D) MS cholinergic neuron accumulation of phospho‐tau in 3xTg (A, B) and 5xFAD (C, D) mice detected by co‐immunofluorescence staining of pTau with ChAT and presented by the ratio of ChAT+ and pTau+ neuron number to ChAT+ neuron number. *n* = 3 Mice per group; scale bar, 20 μm. (E–H) Cholinergic neuron loss in MS of 3xTg (E, F) and 5xFAD (G, H) mice measured by ChAT counting. *n* = 3 Mice per group; scale bar, 100 μm or 20 μm for inset. Unpaired *t‐*test, *t* = 6.753 df = 16, *p *< .01 (F) or *t* = 6.843 df = 16, *p *< .01 (H). **p < .01 versus WT group. Data are presented as mean ± SEM

As MS subset is enriched with cholinergic neurons, we measured the cholinergic tau and the related pathologies. Co‐immunofluorescence staining and quantitative analysis revealed that pTau was increased in MS cholinergic neurons of the AD mice (Figure 2A–D), ∼80% of ChAT‐positive neurons were colocalized with pT205 and pT231 in 10‐month‐old 3xTg (Figure 2A,B) and 5xFAD (Figure 2C,D) mice. Simultaneously, significant ChAT‐positive neuron loss was detected in the MS subset of the AD mice (Figure 2E–H). These data indicate that MS tau accumulation is involved in AD.

### 3.2 Overexpressing hTau in MS induces a time‐dependent spatial memory deficit with dendrite impairment in both MS and hippocampus

**FIGURE 3:**
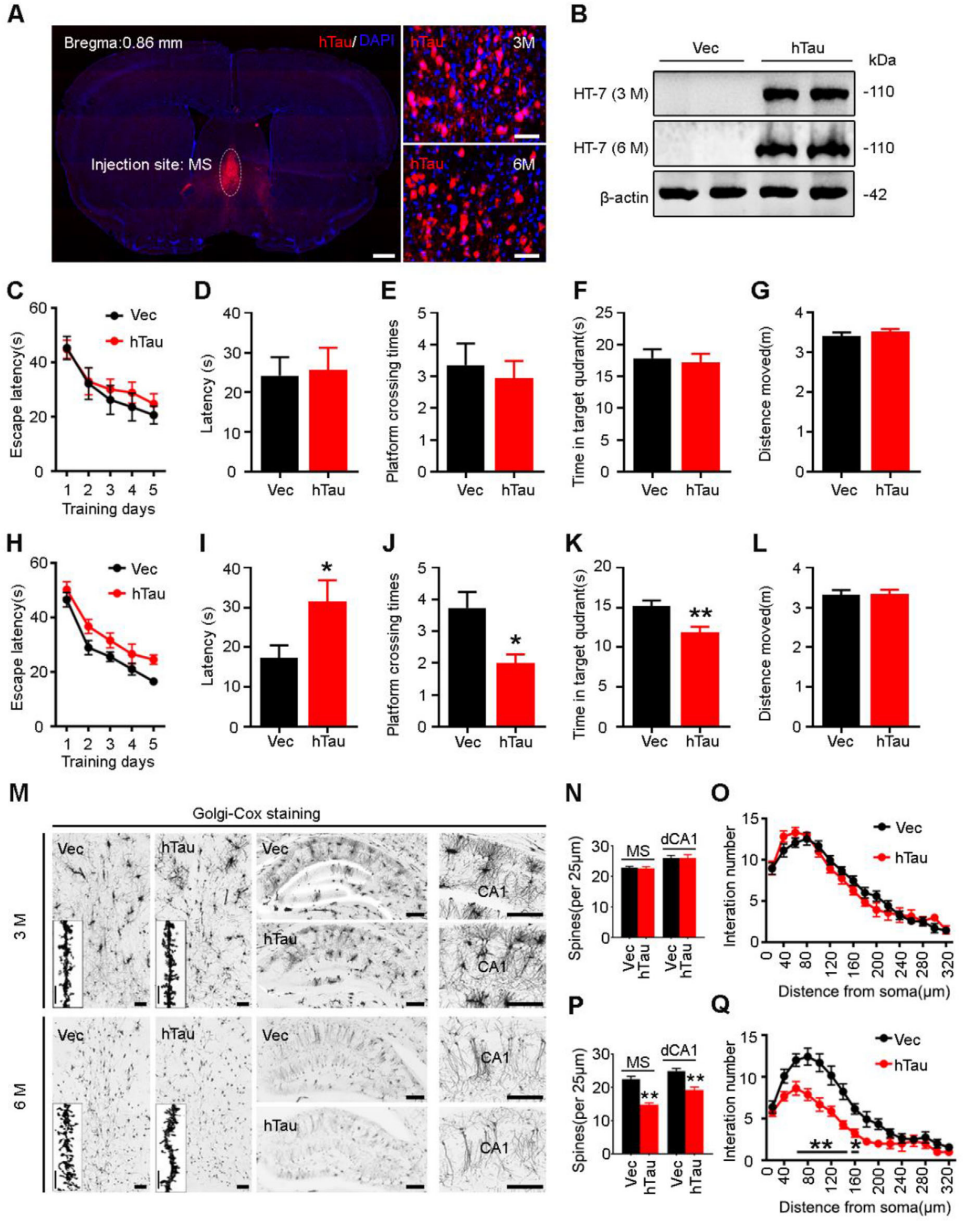
Overexpressing hTau in MS for 6 months but not 3 months induces spatial memory deficit with spine loss in both MS and dCA1. (A and B) Expression of the exogenous hTau detected by confocal imaging (A) and Western blotting (B) at 3 and 6 months after MS infusion of AAV‐CAG‐hTau‐mCherry‐3flag or AAV‐CAG‐vector‐mCherry‐3flag, respectively. HT7 reacts specifically with human tau proteins. Scale bar, left 500 μm, right 50 μm. *n* = 3 Mice per group. (C–G) No spatial cognitive deficit was detected at 3 months after hTau overexpression measured by Morris water maze (MWM) test in learning trial (C, two‐way ANOVA group × days, escape latency: *F*(4, 103) = 0.1551, *p *> .05); and no difference of escape latency to find the platform (D, unpaired *t‐*test, *t* = 0.1874 df = 17, *p *> .05), platform crossing times (E, unpaired *t‐*test, *t* = 0.4942 df = 24, *p *> .05), time in target quadrant (F, unpaired *t‐*test, *t* = 0.3024 df = 23, *p *> .05) and distance moved (G, unpaired *t‐*test, *t* = 1.100 df = 22, *p *> .05) were detected in the probe trails. *n* = 10–15 Mice per group. (H–L) Spatial learning was not changed at 6 months after hTau overexpression shown by the unchanged time to find the platform during 5‐day learning trial (H, two‐way ANOVA group × days, escape latency: *F*(4, 135) = 0.2626, *p *> 0.05); spatial memory deficit was detected at day 7 by removing the platform, shown by an increased time to reach the target area (I, unpaired *t‐*test, *t* = 2.270 df = 24, *p *< .05), less time in target quadrant (K, unpaired *t‐*test, *t* = 3.101 df = 25, *p *< .01), and less crosses in the platform area (J, unpaired *t‐*test, *t* = 2.777 df = 25, *p *< .05) with unchanged moving distance (L, unpaired *t‐*test, *t* = 0.1395 df = 28, *p *> .05). *n* = 13–15 Mice per group. (M–Q) Overexpressing hTau for 6 months reduced MS spine density (P, *n* = 10–15 neurons from three mice per group, unpaired *t‐*test, *t* = 7.652 df = 98, *p *< .01), dCA1 spine density (P, *n* = 10–15 neurons from three mice per group, unpaired *t‐*test, *t* = 4.787 df = 68, *p *< .01), and dCA1 dendritic complexity shown by the decreased branches at 20–140 μm from soma (Q, *n* = 30 neurons from three mice per group, two‐way ANOVA × distance from soma, *F*(15, 328) = 1.831, *p *< .05]; no significant difference in MS spine density (N, *n* = 10–15 neurons from three mice per group, unpaired *t‐*test, *t* = 0.2063 df = 88, *p *> .05), dCA1 spine density (N, *n* = 10–15 neurons from three mice per group, unpaired *t‐*test, *t* = 4.787 df = 68, *p *< .01) or dCA1 dendritic complexity (O, *n* = 30 neurons from three mice per group, two‐way ANOVA × distance from soma, *F*(15, 385) = 1.152, *p *> .05) was detected at 3 months in both MS and hippocampal CA1 measured by Golgi staining. Scale bar, 100 μm or 10 μm (insert graph). *p < .05, **p < .01 versus vector group. Data are presented as mean ± SEM

To verify the causal role of MS tau accumulation in AD‐like spatial memory deficit, we infused stereotaxically AAV‐CAG‐hTau‐mCherry‐3flag or AAV‐CAG‐vector‐mCherry‐3flag (empty vector control) into the MS of 2‐month‐old C57 mice (Figure 3A). Immunofluorescence imaging (Figure 3A) and Western blotting using human tau‐specific antibody HT7 (Figure 3B) were conducted to confirm the expression of hTau in the MS 3 or 6 months after injection. No significant difference among groups was detected in spatial learning (latency to find the hidden platform) during 5‐day training trial by MWM test (Figure 3C,H). In probe trial carried out at day 7, the MS^hTau^ mice showed longer latency, fewer platform crossing times, and less time in the target quadrant than the control mice at 6 months but not at 3 months (Figure 3D–G,I–L), suggesting a time‐dependent spatial memory deficit induced by MS hTau accumulation. No difference of the moved distance was detected among all groups (Figure 3G,L). We also used novel objective recognition test, and no difference was detected among different groups (Figure S1). Additionally, no anxiety‐like behavior was detected by MS hTau accumulation, evidenced by the similar open‐arm entry probability and time spend in open arm; and similar time spent in the center during open‐field test (Figure S2).

By Golgi staining to visualize neuronal spines, we observed that the spine intensity was relatively normal at 3 months both in MS and dCA1 (Figure 3 M,N), while significantly reduced spine density was shown in MS and dCA1 at 6 months after MS‐overexpressing hTau (Figure 3 M,P). Sholl analysis showed that overexpressing hTau for 6 months also significantly reduced dendrite complexity of CA1 neurons (Figure 3O,Q). These data together demonstrate that MS hTau accumulation specifically impairs hippocampus‐dependent spatial memory with dendritic damages in time‐dependent manner.

### 3.3 MS overexpressing hTau induces a time‐dependent increased pathological tau accumulation with neuron loss in MS subset

**FIGURE 4:**
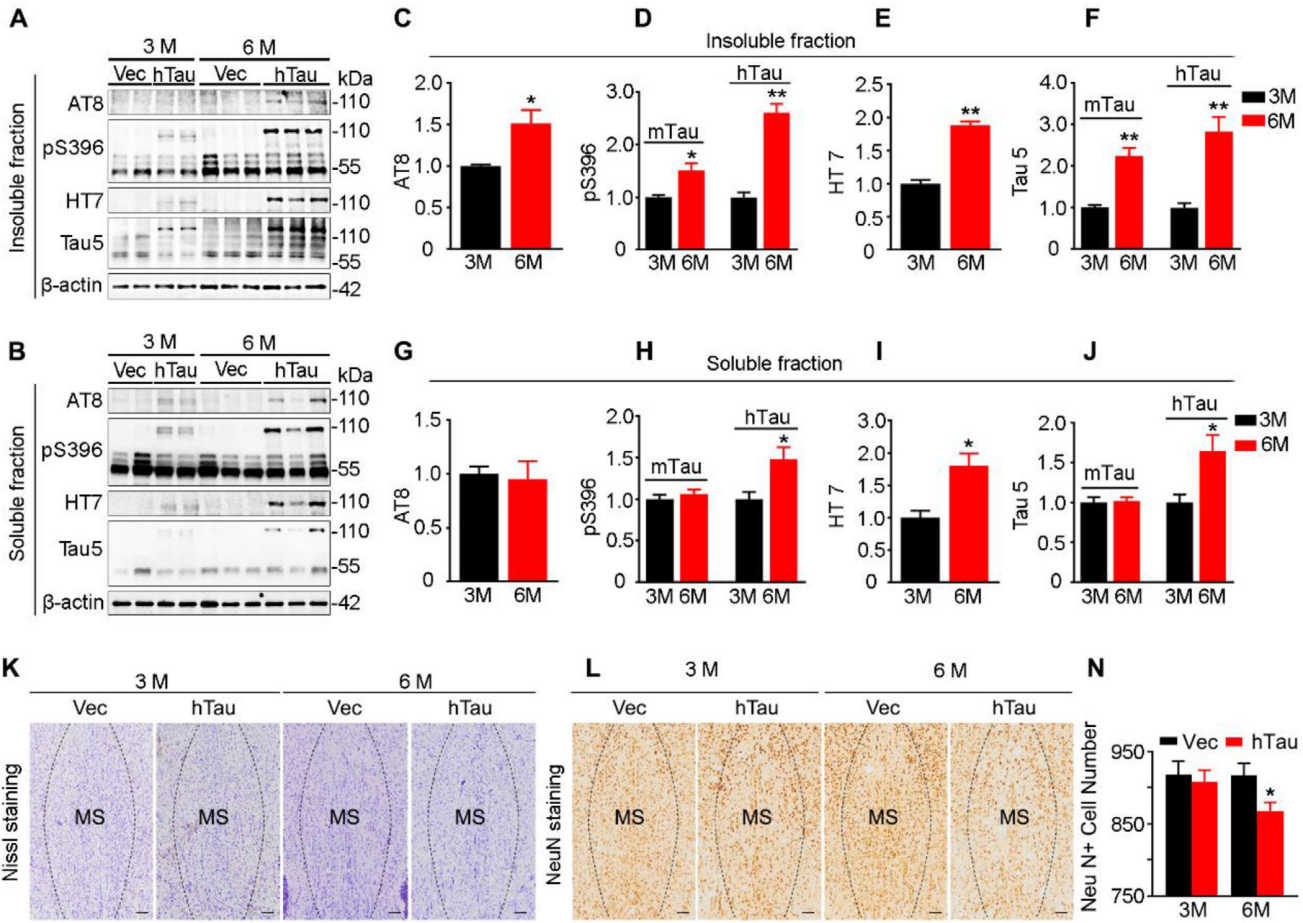
MS overexpressing hTau induces a time‐dependent increased pathological tau accumulation with neuron loss in MS subset. (A–J) Overexpressing hTau in MS induces a time‐dependent tau accumulation in both soluble and insoluble fractions of the MS extracts measured by Western blotting. HT7 reacts with human tau; Tau5, AT8 and pS396 react, respectively, with total tau and phosphorylated tau proteins. *n* = 3–4 Mice per group. For insoluble tau, 3 versus 6 months, unpaired *t‐*test, [AT8]: *t* = 2.588 df = 8, *p *< .05 (hTau); [pS396]: *t* = 3.102 df = 8, *p *< .05 (mTau) or *t* = 7.227 df = 8, *p *< .01 (hTau); [HT7]: *t* = 9.906 df = 8, *p *< .01 (hTau); [Tau5]: *t* = 4.960 df = 8, *p *< .01 (mTau), or *t* = 4.092 df = 8, *p *< .01 (hTau). For soluble tau, 3 versus 6 months, unpaired *t‐*test, [AT8]: *t* = 0.2294 df = 8, *p *> .05 (hTau); [pS396]: *t* = 0.8125 df = 8, *p *> .05 (mTau), *t* = 2.411 df = 8, *p *< .05 (hTau); [HT7]: *t* = 3.176 df = 8, *p *< .05 (hTau); [Tau5]: *t* = 0.2264 df = 6, *p *> .05 (mTau), *t* = 2.604 df = 7, *p *< .05 (hTau). (K–N) Overexpressing hTau in MS induces neuron loss at 6 months but not at 3 months measured by Nissl or NeuN staining. Unpaired *t‐*test, *t* = 0.3950 df = 26, *p *> .05 (3 months) or *t* = 2.334 df = 26, *p *< .05 (6 months). Scale bar, 200 μm. *p < .05, **p < .01 versus vector group. Data are presented as mean ± SEM

To further elucidate the possible mechanisms underlying the time‐dependent memory loss, we first detected pathological tau accumulation by precisely dissected MS subset for Western blotting (Figure 4A,B). In the insoluble fraction, the levels of phospho‐tau at pS396 and AT8, the exogenously overexpressed human tau probed by HT7, and the total tau by Tau5 at high molecular weight (∼110 kDa) were all increased at 6 months compared with 3 months (Figure 4A,C–F). The mouse tau proteins (mTau) at 55∼70 kDa and hyperphosphorylated at pS396 at 6 months compared with 3 months were also significantly increased (Figure 4A,D,F). The level of phospho‐tau at pS396 and total tau was also increased at 3 months after AAV‐hTau infusion compared with the empty vector controls (Figure 4A). Similar results were also detected in the soluble fraction, except for mTau and AT8 (Figure 4B,G–J). Notably, significantly increased AT8‐positive tau (AT8 only reacts with pathologically hyperphosphorylated tau) was only detectable at 6 months in the insoluble fractions of the MS extracts (Figure 4A–C,G), which was coincidently concurrent with the observed spatial memory deficit. By Nissl staining, a significant cell loss in MS was observed at 6 months but not 3 months after overexpressing hTau (Figure 4K). The neuron loss was also detected in MS at 6 months but not 3 months by NeuN staining (Figure 4L,N). These data indicate that overexpressing wild‐type human tau induces neurodegeneration in a time‐dependent manner, which phenocopies the feature of tau pathology observed in AD patients.

### 3.4 MS overexpressing hTau induces a time‐dependent increased cholinergic loss in both MS and hippocampus

**FIGURE 5:**
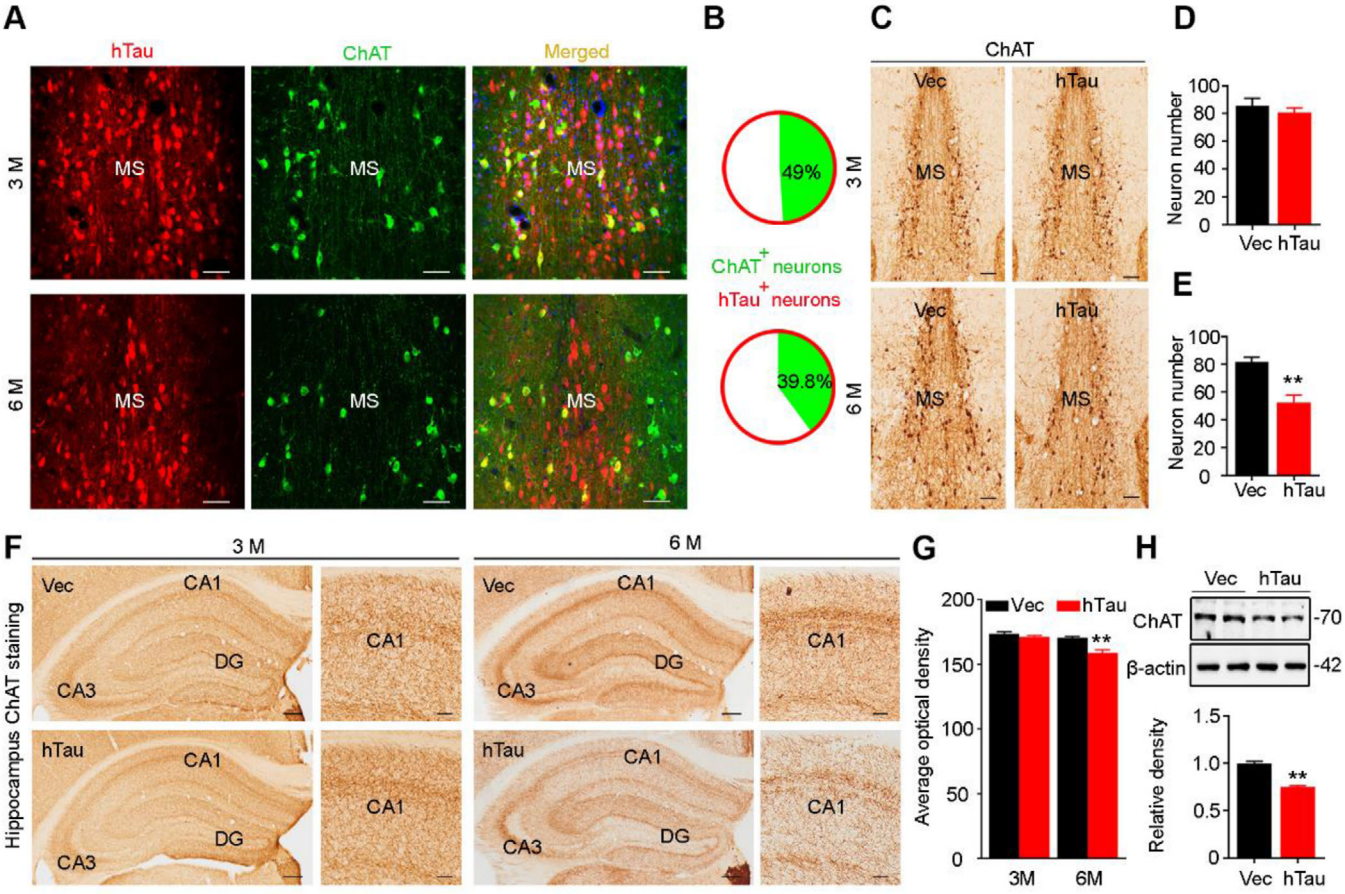
MS overexpressing hTau induces cholinergic loss in both MS and hippocampus at 6 months. (A and B) Colocalization of hTau with ChAT and a reduced cholinergic neuron number were shown at 6 months by co‐immunofluorescence staining of hTau with ChAT in MS. Scale bar, 50 μm. (C–E) Compared with the empty vector, overexpressing hTau for 6 months but not 3 months caused cholinergic neuron loss in MS measured by ChAT immunohistochemical staining. *n* = 3 Mice per group. ChAT+ cell number: unpaired *t‐*test, *t* = 0.7797 df = 16, *p *> .05 (3 months); *t* = 4.592 df = 16, *p *< .01 (6 months). Scale bar, 100 μm. (F and G) Overexpressing hTau for 6 months but not 3 months caused loss of cholinergic projections in hippocampal CA1 measured by ChAT immunohistochemical staining. *n* = 3 Mice per group. ChAT+ fiber intensity: unpaired *t‐*test, *t* = 1.356 df = 10, *p *> .05 (3 months); *t* = 4.576 df = 10, *p *< .01 (6 months). Scale bar, 200 μm or 50 μm in enlarged image. (H) Overexpressing hTau for 6 months decreased ChAT expression in MS measured by Western blotting, and β‐actin was used as a loading control. *n* = 3 Mice per group. Unpaired *t‐*test, *t* = 9.781 df = 4, *p *< .01. *p < .05, **p < .01 versus vector group. Data are presented as mean ± SEM

Given that MS is enriched with cholinergic neurons,^5^, ^23^ we next examined the cell type of tau accumulation by costaining of tau and ChAT. In MS^hTau^ mice, the overexpressed hTau was predominately colocalized with ChAT at both 3 and 6 months (Figure 5A,B). By immunohistochemical staining and quantitative analysis, a significant loss of cholinergic neuron in MS was confirmed at 6 months but not 3 months after hTau overexpression (Figure 5C–E). Meanwhile, the intensity of ChAT+ fibers was remarkably decreased in hippocampal CA1 subset at 6 months but not at 3 months after hTau overexpression (Figure 5F,G). In line with the decreased ChAT+ neurons in MS, reduction of ChAT protein was also detected at 6 months by Western blotting (Figure 5H). Together, these data indicate that overexpressing hTau in MS produces a time‐dependent cholinergic neuron loss in MS and thus reduces cholinergic projections to the hippocampus, such as CA1 subset.

### 3.5 Donepezil rescues spatial memory deficit with improved synaptic transmission and spine density in MS^hTau^ mice

**FIGURE 6:**
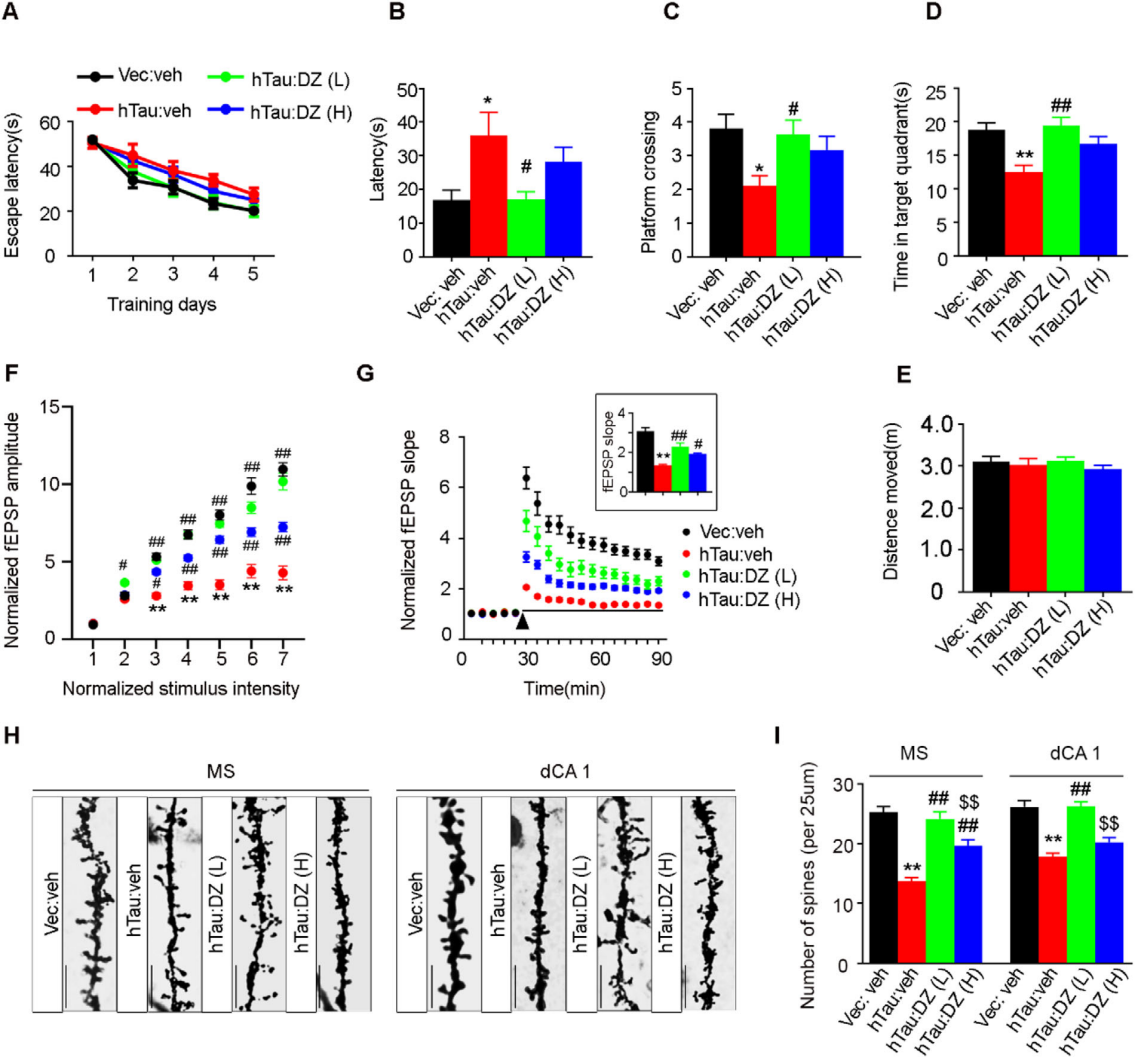
Donepezil rescues MS‐hTau‐induced spatial memory deficit with restoration of synaptic transmission and spine density. (A–D) Donepezil at 1.0 or 2.0 mg/kg/day was injected intraperitoneally at 5 months after MS AAV‐CAG‐hTau‐mCherry‐3flag or AAV‐CAG‐vector‐mCherry‐3flag infusion. After 1 month, the spatial learning and memory were detected by MWM test. During 5‐day learning trial, the mice did not show significant difference in finding the hidden platform (A, two‐way ANOVA group × days, *F*(12, 275) = 0.5554, *p *> .05). In probe trial, the MS^hTau^ mice treated with low‐dose donepezil spent less time to reach the target area (B, one‐way ANOVA, *F*(3, 55) = 5.037, *p *< .01; posttest, *p *< .05 [*p < .05 vs. Vec:veh or #*p *< .05 vs. hTauMS:veh]), more crosses to the platform (C, one‐way ANOVA, *F*(3, 63) = 3.832, *p *= .0138, posttest, *p *< .05 [*p < .05 vs. Vec:veh or #*p *< .05 vs. hTauMS:veh]) and longer staying time in the target quadrant (D, one‐way ANOVA, *F*(3, 63) = 6.026, *p *< .05; posttest, *p *< .05 [**p < .01 vs. Vec:veh or ##*p < *.01 vs. hTauMS:veh]) with unchanged moving distance (E, one‐way ANOVA, *F*(3, 70) = 0.5112, *p *> .05). *n* = 11–18 Mice per group. (F and G) Donepezil at both dosages rescued synaptic transmission in MS^hTau^ mice shown by an increased input–output response (F) and an increased slope of the evoked fEPSP during LTP induction (G), and the increase was still significant at 60 min after high‐frequency stimulation (HFS). *n* = 9–12 Hippocampal slices from five mice in each group, two‐way ANOVA group × intensity, *F*(18, 524) = 16.20, *p *< .01 (F); two‐way ANOVA group × time, *F*(51, 1599) = 9.928, *p *< .01 (G); one‐way ANOVA, *F*(3, 87) = 31.75, *p *< .01 (G, insert graph). *p < .05, **p < .01 versus Vec:veh, #*p *< .05, ##*p *< .001 versus hTauMS:veh. (H and I) Donepezil at both dosages significantly increased spine density in MS, and only low dosage was effective in hippocampus measured by Golgi staining (scale bar, 10 μm). One‐way ANOVA, for MS: *F*(3, 176) = 30.42, *p *< .01; for CA1: *F*(3, 202) = 27.71, *p *< .01. **p < .01 versus Vec:veh, ##*p *< .01 versus hTauMS:veh, $$*p *< .01 versus hTauMS:DZ(L). Data are presented as mean ± SEM

Donepezil, a cholinesterase inhibitor, can effectively maintain Ach activity at cholinergic synapses by inhibiting the breakdown of acetylcholine. To explore whether donepezil could rescue MS hTau‐induced spatial memory deficit, we injected intraperitoneally donepezil at two dosages (1.0 and 2.0 mg/kg per day) for 1 month starting at 5 months after MS AAV‐hTau infusion. No significant difference in latency to find the hidden platform was detected among groups during 5‐day training in MWM test, suggesting no significant change in spatial learning (Figure 6A). During probe trial at day 7, we observed that donepezil treatment at low dosage could improve the hTau‐induced spatial memory loss, evidenced by the decreased latency to the target platform area (Figure 6B), the increased crossings (Figure 6C) and prolong staying time in the target quadrant (Figure 6D) compared with the hTau:veh group. No difference in motor ability was found among the groups (Figure 6E). These data indicate that low‐dosage donepezil could efficiently rescue the MS hTau‐induced spatial memory deficit.

The hippocampal Schaeffer collateral (SC) to CA1 synapses are among the mostly studied for hippocampus synaptic plasticity,^24^ a widely recognized cellular model for spatial cognitive capacity.^25^ To investigate whether donepezil benefits this form of hippocampal plasticity, we performed electrophysiological recording in hippocampal slice. Compared with the vehicle control groups, administration of donepezil at both dosages (1 and 2 mg/kg/day) for 1 month rescued the MS hTau‐induced inhibition of synaptic transmission (Figure 6F) and LTP in hippocampal CA1 subset (Figure 6G). These beneficial effects were more predominant at low dose of donepezil group than high‐dose group (Figure 6F,G). By Golgi staining, we also found that low dose of donepezil was more efficient than high dose in restoring the spine density of both MS and hippocampal CA1 subsets after MS hTau‐overexpression for 6 months (Figure 6H,I).

### 3.6 Donepezil rescues cholinergic loss with removal of tau proteins in MS^hTau^ mice

**FIGURE 7:**
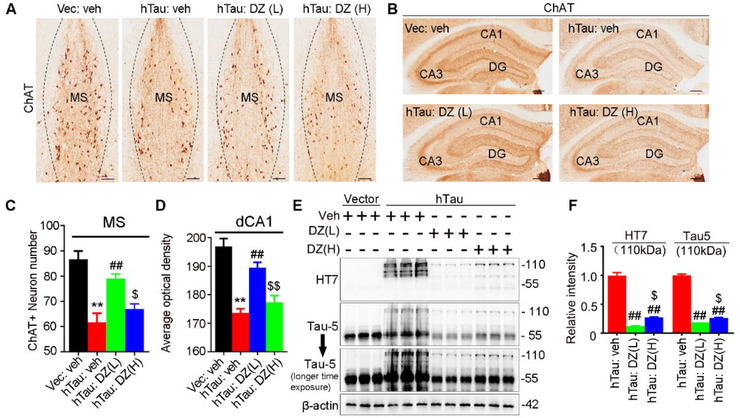
Low‐dose donepezil more prominently rescues MS‐hTau‐induced cholinergic loss with removal of tau proteins. (A–D) Both high‐ and low‐dose donepezil restored ChAT staining, and the effect of low dose was more prominent than high dose in MS (A and C, scale bar = 100 μm) and hippocampus (B and D, scale bar = 100 μm) measured by ChAT immunohistochemistry staining. One‐way ANOVA group, *F*(3, 32) = 16.08, *p *< .01 (**p < .01 vs. Vec:veh, ##*p *< .01 vs. hTau:veh, $*p *< .05 vs. hTau:DZ(L)) (C) or *F*(3, 52) = 25.52, *p *< .01 (**p < .01 vs. Vec:veh, ##*p *< .01 vs. hTau:veh, $$*p *< .01 vs. hTau:DZ(L)) (D). (E and F) Low‐dose donepezil more significantly decreased human tau proteins (HT7 and Tau‐5 at ∼110 kDa band) than high dose in MS^hTau^ mice measured by Western blotting. One‐way ANOVA group, *F*(2, 6) = 254.9, *p *< .01 (HT7); *F*(2, 6) = 802.1, *p *< .01 (Tau‐5). #*p *< .05, ##*p *< .01 versus hTauMS:veh, $*p *< .05. HT7 specifically reacts with the exogenously expressed hTau, and Tau‐5 reacts with total tau proteins. Data are presented as mean ± SEM

To explore how donepezil may improve the MS hTau‐induced spatial memory deficits, we first measured cholinergic neurons by ChAT staining. We found that both ChAT+ neuron number and the intensity of ChAT+ fibers were enhanced in donepezil‐treated MS‐hTau mice and more prominent effect was shown at low dose, when compared with the hTau:veh group (Figure 7A,C). Meanwhile, donepezil treatment at low dose for 1 month also restored the MS hTau‐induced reduction of cholinergic projections to the hippocampal CA1 subset (Figure 7B,D). We also found that donepezil at both low and high doses decreased tau proteins (especially at molecular mass of ∼110 kDa) in the MS extracts measured by Western blotting using HT7 and Tau5, and a much more significant reduction was shown by low dose (Figure 7E,F). These data suggest that donepezil can attenuate MS hTau‐induced cholinergic lesions on the MS–hippocampus pathway with the mechanism involving reducing tau‐load, and low‐dose donepezil is much more efficient than high dose.

### 3.7 Donepezil modifies protein network in MS^hTau^ mice

**FIGURE 8:**
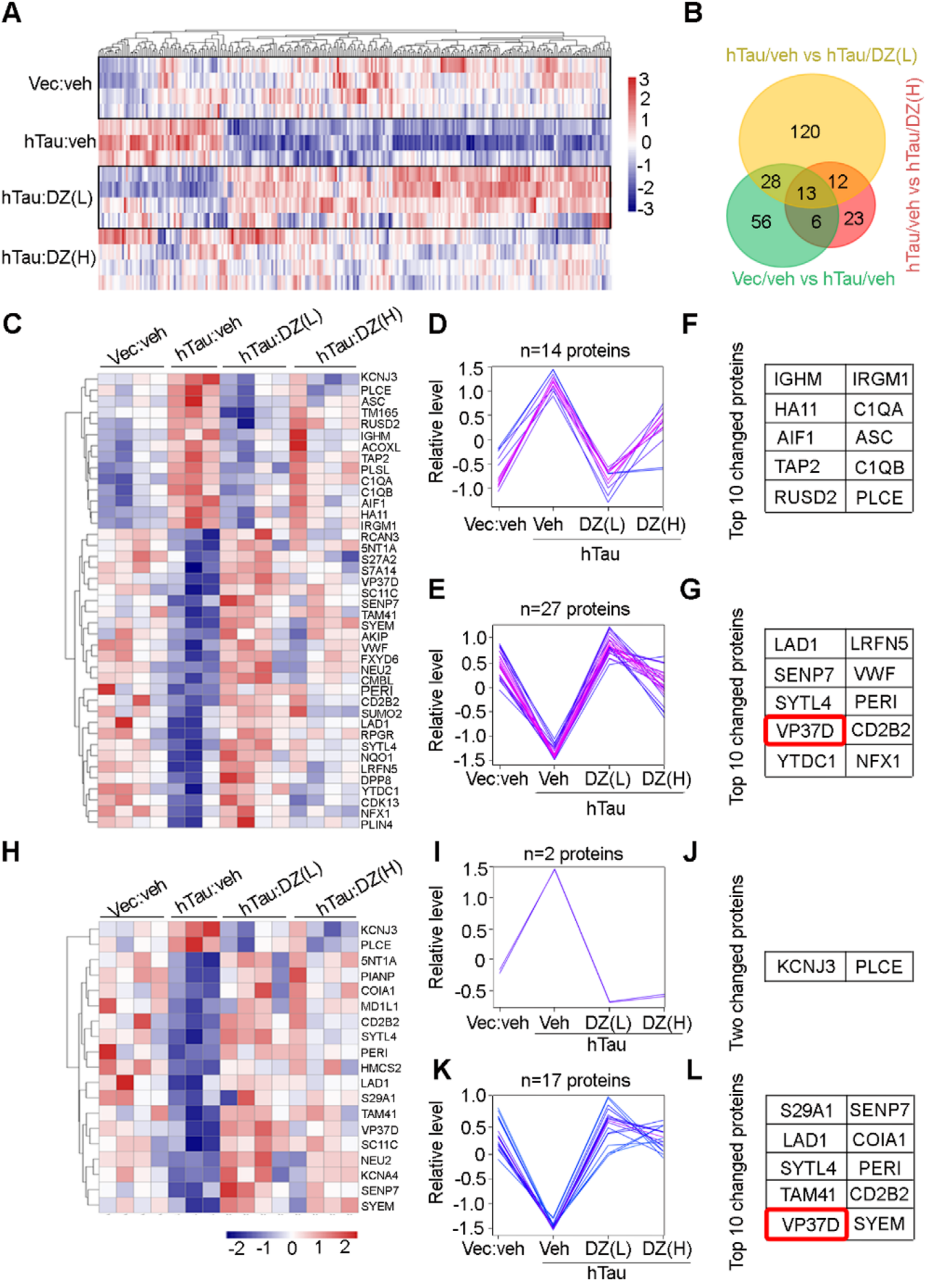
Donepezil modifies protein network in MS^hTau^ mice. (A) Heatmap showing dysregulated MS proteins induced by hTau accumulation and its modification by donepezil treatment. Ratios ≥1.2 or ≤0.83. (B) Venn diagram represents the number of differentially expressed proteins (DEPs) identified among the groups. (C–G) Forty‐one DEPs between vec:veh versus hTau:veh and hTau:veh versus hTau:DZ (low dose) (C), their changing trends (D, F), and the top 10 changed proteins in D and F (E, G). (H–L) Nineteen DEPs between vec:veh versus hTau:veh and hTau:veh versus hTau:DZ (high dose) (H), their changing trends (I, K), and the two or top 10 changed proteins in I and K (J, L)

To further investigate the molecular mechanisms underlying the beneficial effects of donepezil on MS hTau accumulation, we precisely dissected MS region and performed an unbiased proteomic analysis among the groups. Together, 103, 173, and 54 DEPs were detected in Ctrl versus MS^hTau^, MS^hTau^ versus MS^hTau^/low‐dose donepezil, and MS^hTau^ versus MS^hTau^/high‐dose donepezil groups, respectively (Figure 8A,B).

Further comparative analysis revealed 41 common DEPs between Ctrl versus MS^hTau^ and MS^hTau^ versus MS^hTau^/low‐dose donepezil. Among them, 14 proteins were increased in MS^hTau^ mice but decreased in the MS^hTau^/donepezil. For the top 15 most significantly changed proteins, ∼33% of them were reversed by low‐dose donepezil (Table S1). The top 10 proteins among them were enriched in the pathway of immunity and inflammation, that is immunoglobulin heavy constant mu (IGHM), immunity‐related GTPase family M protein 1 (IRGM1), complement C1q subcomponent subunit A (C1QA), complement C1q subcomponent subunit B (C1QB), allograft inflammatory factor 1 (AIF1), antigen peptide transporter 2 (TAP2), apoptosis‐associated speck‐like protein containing a CARD (ASC) and H‐2 class I histocompatibility antigen, D‐B alpha chain (HA11); and in the pathway of transferase activity (1‐acyl‐sn‐glycerol‐3‐phosphate acyltransferase epsilon, PLCE); and RNA binding (RNA pseudouridylate synthase domain‐containing protein 2, RUSD2). There were 27 proteins decreased in MS^hTau^ mice but increased in MS^hTau^/donepezil (Figure 8C,D). For the top 15 downregulated proteins, ∼53% were reversed by low‐dose donepezil (Table S2). The top 10 proteins among them were associated with anchoring and adhesion, that is, ladinin‐1 (LAD1), leucine‐rich repeat and fibronectin type‐III domain‐containing protein 5 (LRFN5), and von Willebrand factor (VWF); posttranslational modification (sentrin‐specific protease 7, SENP7); exocytosis (synaptotagmin‐like protein 4, SYTL4); intermediate filament cytoskeleton organization (perilipin, PERI); autophagy (vacuolar protein sorting‐associated protein 37D, VP37D); immunity (transcriptional repressor NF‐X1, NFX1); and others, such as CD2 antigen cytoplasmic tail‐binding protein 2 (CD2B2) and YTH domain‐containing protein 1 (YTDC1) (Figure 8E).

When the common DEPs were analyzed between Ctrl versus MS^hTau^ and MS^hTau^ versus MS^hTau^/high‐dose donepezil, we found that G protein‐activated inward rectifier potassium channel 1 (KCNJ3) and PLCE were significantly increased in MS^hTau^, but decreased in the MS^hTau^/donepezil (Figure 8H–J). The rest 17 proteins showed opposite alterations (Figure 8K). Among them, the top 10 changed proteins were associated with influx and efflux of nucleosides, such as equilibrating nucleoside transporter 1 (S29A1); posttranslational modification (SENP7); anchoring and adhesion (LAD1); exocytosis (SYTL4); intermediate filament cytoskeleton organization (PERI); mitochondrial function (phosphatidate cytidylyltransferase, TAM41); autophagy (VP37D); and others, such as collagen alpha‐1(XVIII) chain(COAI1), CD2 antigen cytoplasmic tail‐binding protein 2 (CD2B2), and probable glutamate‐tRNA ligase (SYEM).

There were 13 common DEPs among Ctrl versus MS^hTau^, MS^hTau^ versus MS^hTau^/low‐dose, and MS^hTau^ vs MS^hTau^/high‐dose donepezil. KCNJ3 and PLCE were increased in MS^hTau^, and decreased in MS^hTau^/donepezil (Figure 8J). SENP7, VP37D, SYTL4, LAD1, NEU2 (vasopressin‐neurophysin 2‐copeptin), CD2B2, PERI, TAM41, SYEM, SC11C (signal peptidase complex catalytic subunit SEC11C), and 5NT1A (cytosolic 5′‐nucleotidase 1A) were decreased in MS^hTau^, but increased in the MS^hTau^/donepezil (Figure 8G,L).

Given that low‐dose donepezil was more efficient than high‐dose donepezil in reducing MS hTau, we analyzed DEPs between MS^hTau^/low‐dose and MS^hTau^/high‐dose donepezil groups and compared them with the significantly changed proteins in Ctrl versus MS^hTau^ groups. The results (Table 1) showed that among various DEPs, VP37D was significantly decreased in hTau group as compared with control, and donepezil could restore the level, and donepezil at low dosage even significantly increased the level of VP37D. VP37D is a component of ESCRT‐I complex closely associated with autophagy.^26^, ^27^ These data imply that the increased VP37D may be involved in donepezil‐induced tau reduction.

**TABLE 1:** VP37D level measured by medial septum (MS) proteomics

Protein	Grouping			
	Vec:veh(*n* = 4)	MS^hTau^:veh(*n* = 3)	MS^hTau^:DZ(L) (*n* = 4)	MS^hTau^:DZ(H) (*n* = 4)
VP37D	1.007 ± 0.03513	0.7632 ± 0.07199	1.191 ± 0.03077	0.9797 ± 0.03644

*Note*: Data are presented as mean ± SEM. One‐way ANOVA, *F*(3, 11) = 15.81, *p *= .0003, Tukey's multiple comparisons test, Vec:veh versus MS^hTau^:veh: *p *= .0110; MS^hTau^:veh versus MS^hTau^:DZ(L): *p *= .0001; MS^hTau^:veh versus MS^hTau^:DZ(H): *p *= .0230; MS^hTau^:DZ(L) versus MS^hTau^:DZ(H): *p *= .0169.

## 4 DISCUSSION

Cholinergic impairment plays a crucial role in AD pathogenesis, therefore, preserving cholinergic function has been one of the currently approved two classes of drugs by FDA (food and drug administration). Spatial memory loss is the early symptom of AD, and hippocampus formation takes charge of spatial cognitive functions. In the brain, the MS subset provides the primary source of cholinergic input to the hippocampus and plays a crucial role in promoting hippocampus‐dependent learning. Hippocampal theta‐rhythm oscillation is required for memory acquisition,^28^ and this oscillation is modulated by MS activity.^20^, ^29^, ^30^ Removing MS cholinergic projections by deleting vesicular acetylcholine transporter (vAChT), which is required for acetylcholine release, significantly impairs hippocampus‐dependent working memory.^31^, ^32^ Recent studies demonstrated that hippocampal tau accumulation at different subsets or different cell types induces spatial memory deficits^15^, ^16^, ^33^, ^34^; however, whether MS would also be vulnerable to tau accumulation and thus affect spatial cognitive functions is elusive.

In the present study, we observed abnormal tau accumulation in MS subset of two AD transgenic mouse models, 3xTg and 5xFAD. To explore the causal role of MS tau accumulation, we overexpressed human wild‐type full‐length tau (hTau) in MS to mimic the AD‐like hTau accumulation because no mutation of tau gene has been identified in AD patients to date.^35^ A time‐dependent accumulation of the hyperphosphorylated tau was detected in the MS after overexpressing hTau. Interestingly, we observed more significantly increased tau phosphorylation at AT8 and pS396 epitopes in the insoluble and soluble fractions, respectively. The mechanism for this phenomenon is currently not understood. It could be attributed to the upregulated hTau expression itself or the activated kinase(s) that were more effective to AT8 or Ser396. We also found that overexpressing hTau in MS induced time‐dependent spatial memory deficits with inhibition of synaptic transmission and dendrite impairment. Simultaneously, overexpressing hTau caused cholinergic neuron loss and weakened cholinergic projections from MS to the hippocampus in a time‐dependent manner. These findings indicate that the cholinergic MS–hippocampus pathway is essential for spatial cognition, because progressive loss of MS cholinergic neurons and insufficiency of cholinergic inputs to the hippocampus can cause spatial memory deficit.

Cholinergic signaling regulates hippocampal synaptic functions and spatial memory. In the MS^hTau^ mice, we also detected a significant inhibition of synaptic transmission and LTP induction in hippocampal SC to CA1 synapses. This result was consistent with the finding that optogenetic activation of septal cholinergic input allows the expression of distinct time‐dependent forms of hippocampal plasticity.^36^ In hippocampus, AChRs are located both pre‐ and postsynaptically. Activation of septal cholinergic inputs could increase presynaptic release via α7 nAChRs (nicotinic AChRs) and enhance postsynaptic function by M1 mAChRs (muscarinic AChRs).^36^ Thus, both distorted pathways can contribute to the inhibition of synaptic transmission and LTP induction in the hippocampus of MS^hTau^ mice. A previous study showed that selective knockout vChAT in the hippocampus damaged working memory with no significant impairment on spatial memory,^31^ suggesting that the MS‐overexpressing hTau may induce spatial cognitive deficit with more complex mechanisms in addition to cholinergic damages.

We detected beneficial effects of donepezil on hippocampal synaptic transmission, plasticity and spatial memory in MS^hTau^ mice. These may, at least in part, attribute to the acetylcholinesterase inhibition by donepezil, which has been approved for clinical treatment of AD. In addition, we observed that donepezil at low dose could preserve cholinergic neuron numbers in MS and their projections to hippocampus, which may also contribute to the improved spatial memory of MS^hTau^ mice. Unexpectedly, we also found that donepezil could remarkably reduce tau accumulation in the MS^hTau^ mice. In AD, the ubiquitin‐proteasome system is disrupted,^37^, ^38^ resulting in a build‐up of intracellular tau. Tau is also a target of autophagy,^39^ deficits in the autophagy processing could increase the intracellular tau, leading to tau aggregation and accumulation. Meanwhile, tau is also found to disrupt IST‐regulated ESCRT‐III complex formation to repress autophagy flux, and in turn exacerbates tau aggregation.^40^ Therefore, tau accumulation and autophagy inhibition could form a vicious cycle to progressively aggravate the AD pathologies.

To explore how hTau impaired MS, we did a proteomics analysis by precisely dissecting the MS subset after overexpressing hTau. The proteomics in MS extracts revealed 103 DEPs in Ctrl versus MS^hTau^ mice. The top 10 upregulated proteins were, respectively, associated with immunity, inflammation, synaptic transmission and so on, while the top 10 downregulated proteins were primarily concentrated in anchoring and adhesion, posttranslational modifications, exocytosis, lipid metabolism, autophagy, and so on. To some extent, these unbiased proteomic data provide new clues for the potential molecular mechanisms underlying tau‐impaired cholinergic deficits, in addition to the currently known AChE^17^ and HDAC2.^19^ Whether and how the above molecules are provoked by MS‐hTau accumulation and how they may contribute to the hTau‐induced time‐dependent cholinergic lesions deserve further investigation. Given that MS consists of three major neuronal types, that is, cholinergic, GABAergic, and glutamatergic neurons,^41^, ^42^ the comparison among cholinergic, GABAergic, and glutamatergic neurons may be conducted in future to clarify the vulnerability of MS cholinergic neurons to hTau accumulation.

The proteomic data revealed that both low‐ and high‐dose donepezil modified hTau‐impaired pathways of posttranslational modification, anchoring and adhesion, exocytosis, adipocyte lipid metabolism, and autophagy, indicating the multiple targets and novel functions of donepezil in addition to its traditional function of acetylcholinesterase inhibition. Interestingly, we found that low‐dose donepezil showed much more significant effect on reducing tau accumulation. The mechanism underlying this selective disease‐modifying effect of donepezil is currently not clear, the following evidence may be involved. First, low‐dose donepezil restored more dysregulated MS proteins than high‐dose in hTau mice (41 vs. 19 DEPs). Furthermore, low‐dose donepezil significantly modified the top 15 (∼33%) upregulated DEPs in hTau group, while high dose had no effect on these proteins. In terms of tau‐modified signal pathways, only low‐dose but not high‐dose donepezil exhibited strong effect on restoring immunity and inflammation, evidenced by the recovery of C1QA, IRGM1, AIF1, HA11, and IGHM. Increasing C1QA could promote synaptic pruning by microglia and cause memory loss in AD model.^43^ An increased plasma IGHM was negatively correlated with cognitive status in both AD and MCI patients and IGHM could clear Aβ.^44^, ^45^ These data together strongly suggest that low‐dose donepezil ameliorates tau toxicity with modification of inflammation pathway, which is supported by the previous report that donepezil exhibits anti‐inflammation effect on high‐fat diet mouse model.^46^ Additionally, low‐dose donepezil even increased VP37D, a component of ESCRT‐I,^26^, ^27^ suggesting autophagy improvement by donepezil. No matter what may be the mechanism, the novel function of donepezil in reducing tau suggests its potential disease‐modifying role in addition to the symptom improvement in AD treatment.

In the clinic, an “optimal’’ treatment of AD with cholinesterase inhibitor is currently lacking. Some physicians adopt strategy to escalate the dose as quickly as possible and maintain patients on the highest recommended doses, while others prefer staying on lower doses until the patients deteriorate with more significant symptoms. Donepezil can inhibit cholinesterase in dose‐dependent manner.^47^ Given that the low‐dose donepezil exerted unexpectedly superior effects to high dose in removing tau, rescuing cholinergic loss, hippocampal synaptic plasticity, and spatial memory in MS^hTau^ mice, it could be recommended for those patients with severer tau pathologies. On the other hand, high‐dose donepezil might be more suitable for patients with severe mitochondrial dysfunction because it exhibited beneficial effect on mitochondrial proteins, such as TAM41 and SENP7.

As MS subset has three major neuronal types, that is, cholinergic, glutamatergic, and GABAergic neurons, further neurotype‐specific hTau overexpression and/or single‐cell proteomics will definitely reveal more precisely the effects of hTau on cholinergic neuron and its projection/circuitry. Verification of the identified DEPs also deserves further investigation.

Together, we found that MS tau accumulation not only induces cholinergic damages in MS but also impairs its hippocampal projections, by which it causes spatial memory deficit as observed at early stage of AD. Notably, low‐dose donepezil improves the spatial cognitive functions with significantly decreased tau‐load and cholinergic loss in MS^hTau^ mice. This novel therapeutic effect may extend the application of donepezil beyond simple symptom amelioration to potential disease modification, and provide new information for optimizing dosing in donepezil treatment on AD.

## CONFLICT OF INTEREST

The authors declare that there is no conflict of interest.

## ETHICS STATEMENT

No patient data or clinical studies were included in this manuscript. All animals involved in our research were cared under the provision and suggestions of the Chinese Experimental Animals Administration Legislation. The experiment procedures were approved by the Animal Protection and Use Committee of Huazhong University of Science and Technology.

## AUTHOR CONTRIBUTIONS

Dongqin Wu, Ying Yang, and Jianzhi Wang designed the research. Dongqin Wu, Di Gao, Haitao Yu, Guilin Pi, Rui Xiong, Huiyang Lei, Xin Wang, Huilin Yu, Yang Gao, Ting He, Tao Jiang, Fei Sun, Jingfen Su, Guoda Song, and Wenju Peng carried out the experiments. Di Gao, Enjie Liu, and Jinwang Ye contributed to experimental methods. Dongqin Wu, Haitao Yu, Ying Yang, and Jianzhi Wang analyzed the data by double‐blind method. Dongqin Wu, Ying Yang, and Jianzhi Wang wrote the paper. Jianzhi Wang provided project administration.

## Supporting information

Supporting Information

## Data Availability

Data supporting the results of this study are available from the corresponding author.
