# Erratum for the article “Medial septum tau accumulation induces spatial memory deficit via disrupting medial septum‐hippocampus cholinergic pathway” by Wu et al.

**DOI:** 10.1002/ctm2.70234

**Published:** 2025-02-16

Dear editor,

We are writing this letter to apply for an erratum of our published paper by Wu et al. We sincerely apologize for the mistakes and deeply regret any inconvenience this may have caused you.

Original version of Figure 5

The red block highlights the incorrect image of Vector control group.

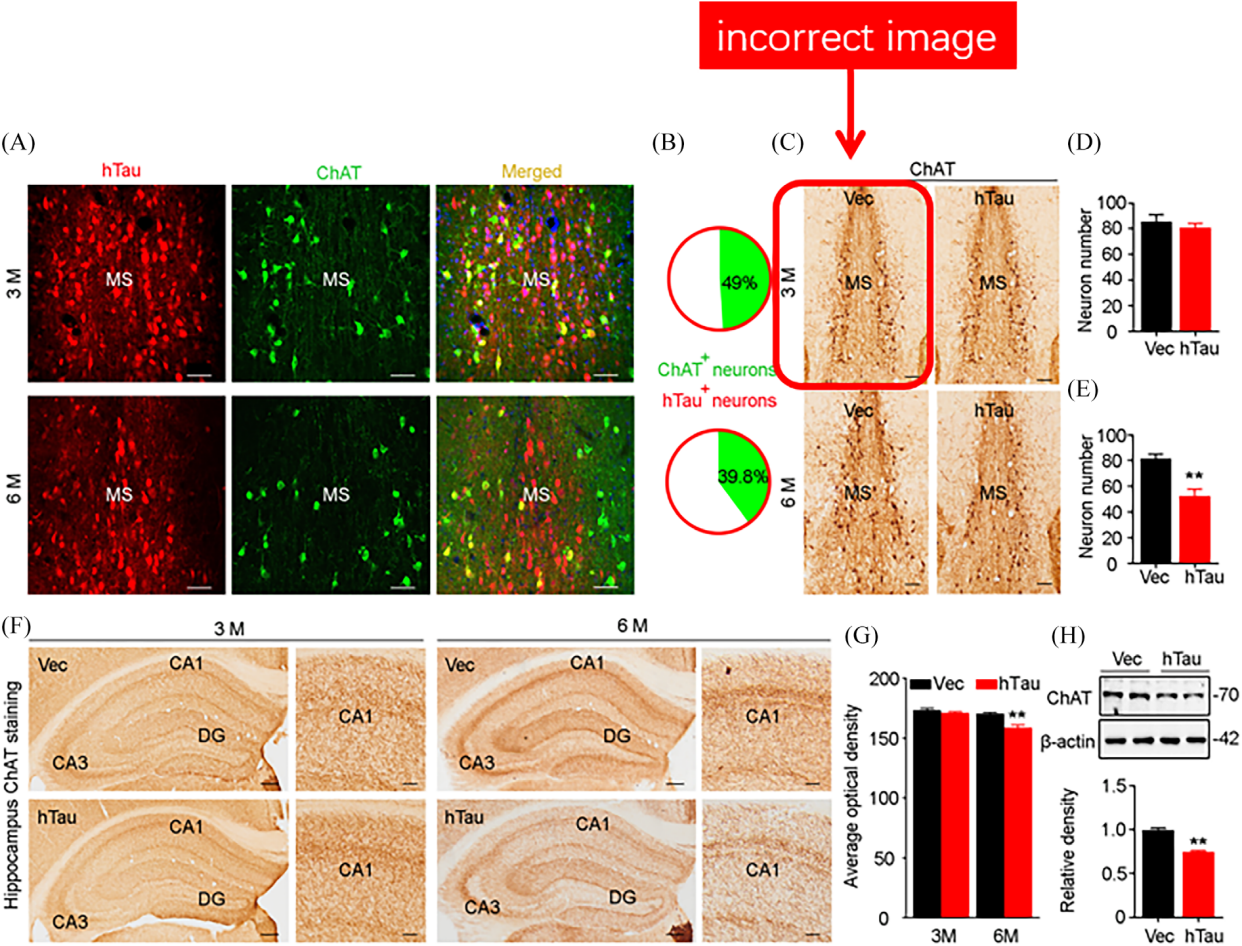

The new version of Figure 5

A correct image of the vector control group (3 M) is included in Figure 5C.

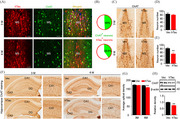

The amended Figure 5

The reason for correction:

I recently discovered an error in Figure 5 while reviewing my paper. In the original version of Figure 5C, for 3 months after virus injection (upper panel), the image from Vector control group was inadvertently replaced by hTau group. Upon reviewing the original data, we realized that the mistake was made by inadvertently duplicated the layers during figure preparation. We have now included the correct image of Vector control group in the amended Figure 5. There were no significant differences in ChAT+ signals between the vector and overexpressing hTau groups (3 months). The experimental conclusions remain unchanged by this revision; therefore, no adjustments to the article's text are necessary.

Thank you for your consideration. We look forward to your kind response.

Sincerely yours,

Dr. Dongqin Wu

Department of Pathophysiology, School of Basic Medicine, Key Laboratory of Education Ministry of China/Hubei Province for Neurological Disorders, Tongji Medical College, Huazhong University of Science and Technology, Wuhan, China

