# Supplementary material for: Medial septum tau accumulation induces spatial memory deficit via disrupting medial septum–hippocampus cholinergic pathway
Source: Clin Transl Med. 2021 May 28;11(6):e428. doi: 10.1002/ctm2.428 (PMC8161512; doi:10.1002/ctm2.428)
Supplement: Supplementary file 1 — Supporting Information [file CTM2-11-e428-s001.pdf]

Supplementary figures

sFigure 1

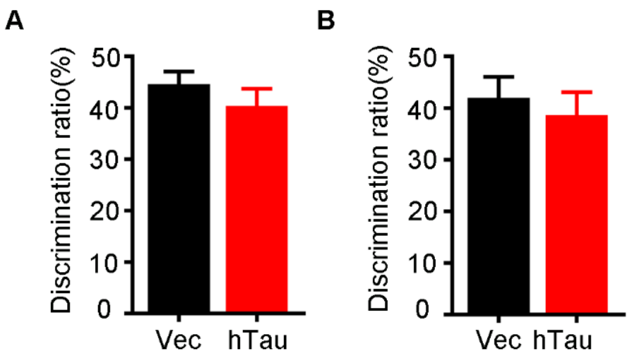

sFigure 1. Overexpressing hTau in MS does not affect novel object recognition memory. (**A**) 3 months after MS overexpression of AAV-hTau, n = 10-15 mice per group, unpaired *t* test, t = 0.9754 df = 23, $P > 0.05$. (**B**) 6 months after MS overexpression of AAV-hTau, n = 10-15 mice per group, unpaired *t* test, t = 0.5499 df = 27, $P > 0.05$. Data were presented as mean ± SEM.

sFigure 2

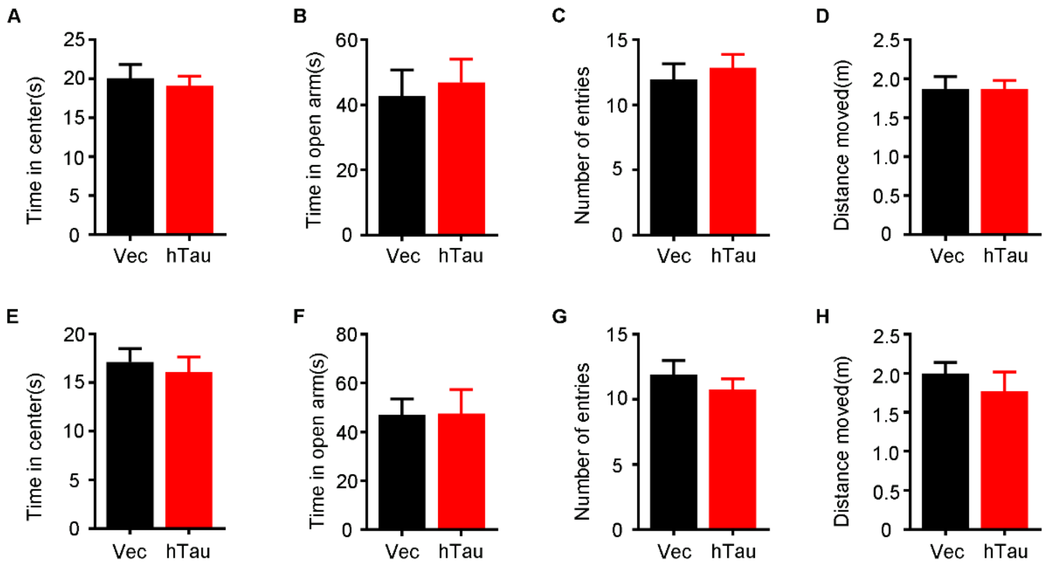

sFigure 2. Overexpressing hTau in MS does not induce anxiety-like behavior in MS^{hTau} mice. Open field test (A, E) and elevated plus maze test (B-D, F-H) were performed. No difference of time in center (A, E), time in open arm (B, F) and open arm entry (C, G) were shown between MS^{vec} and MS^{hTau} mice. For 3 months after MS hTau overexpression, n = 10-15 mice per group, unpaired *t* test, t = 0.4826 df = 24, $P > 0.05$ (A, time in center); or t = 0.3933 df = 22, $P > 0.05$ (B, time in open arm); or t = 0.5758 df = 21, $P > 0.05$ (C, open arm entry); or t = 0.005621 df = 25, $P > 0.05$ (D, distance moved). For 6 months after MS hTau overexpression, n = 10-15 mice per group, unpaired *t* test, t = 0.5122 df = 22, $P > 0.05$ (E, time in center); or t = 0.04848 df = 20, $P = 0.9618$ (F, time in open arm); or t = 0.8646 df = 19, $P > 0.05$ (G, open arm entry); or t = 0.8440 df = 25, $P > 0.05$ (H, distance moved). Data were presented as mean ± SEM.

Supplementary tables

sTable 1

The top 15 up-regulated differentially expressed proteins (DEPs) in Vec:veh vs MS^{hTau} group

Protein	Grouping			
	Vec:veh (n=4)	MS^{hTau}:veh (n=3)	MS^{hTau}:DZ(L) (n=4)	MS^{hTau}:DZ(H) (n=4)
STAT1	0.867723±0.028608	1.127335±0.039638	-	-
AIF1	0.891054±0.043824	1.172083±0.040145	0.941832±0.030447	-
IFIT3	0.873289±0.047846	1.162859±0.028778	-	-
C1QA	0.857073±0.04464	1.156626±0.031093	0.91963±0.01657	-
CLD11	0.918516±0.035997	1.245412±0.131718	-	-
TPSN	0.829519±0.017523	1.14166±0.097453	-	-
GBP4	0.866065±0.055694	1.221984±0.087853	-	-
HA11	0.857359±0.016084	1.211533±0.076491	0.906532±0.081473	-
GFAP	0.805562±0.021275	1.221036±0.132556	-	-
IIGP1	0.810976±0.037984	1.245658±0.144553	-	-
IRGM1	0.824163±0.036841	1.293765±0.078868	0.879048±0.090735	-

IGHA	0.681882±0.023736	1.08046±0.08342	-	-
PSB8	0.773077±0.022639	1.228403±0.052214	-	-
S10A6	0.786228±0.031363	1.257172±0.228963	-	-
IGHM	0.574758±0.092829	1.663775±0.260839	0.699626±0.173127	-

sTable 2

The top 15 down-regulated differentially expressed proteins (DEPs) in Vec:veh vs MS^{hTau} group

Protein	Grouping			
	Vec:veh (n=4)	MS^{hTau}:veh (n=3)	MS^{hTau}:DZ(L) (n=4)	MS^{hTau}:DZ(H) (n=4)
KLDC4	1.356578±0.078396	0.683064±0.147207	-	-
S29A1	1.197628±0.12626	0.636127±0.120676	-	1.046025±0.053886
LAD1	1.132309±0.126257	0.718013±0.111504	1.089028±0.019657	0.990153±0.02422
LRFN5	1.101031±0.052344	0.71772±0.136019	1.146596±0.081776	-
SENP7	0.96118±0.034541	0.662861±0.094933	1.285554±0.128007	1.00612±0.085685
VWF	1.095346±0.065769	0.777465±0.062261	1.090642±0.058394	-
CPTP	1.112783±0.04531	0.811406±0.089863	-	-
SYTL4	1.022702±0.046996	0.76104±0.042287	1.154798±0.044964	1.00172±0.038575
PERI	1.05395±0.098073	0.789833±0.018686	1.077725±0.040215	1.02595±0.033756
MNARL	1.121862±0.066664	0.846557±0.06295	-	-
VP37D	1.00711±0.035125	0.763247±0.071989	1.190753±0.030767	0.979701±0.036441
CD2B2	1.040066±0.083526	0.791044±0.025873	1.099758±0.039808	1.016892±0.059873

OS9	1.135266±0.036994	0.86383±0.077718	-	-
AGRP	1.071368±0.056671	0.820164±0.030231	-	-
HMCS2	1.092863±0.033541	0.843392±0.008601	-	1.028653±0.050647
